# Red cell distribution width positively correlates with 10-year risk of cardiovascular disease among people with type 1 diabetes as assessed by the Steno Type 1 Risk Engine

**DOI:** 10.1007/s00592-025-02615-y

**Published:** 2025-11-24

**Authors:** Dariusz Naskret, Stanislaw Pilacinski, Pawel Niedzwiecki, Michal Kulecki, Dorota Zozulinska-Ziolkiewicz

**Affiliations:** Department of Internal Medicine and Diabetology, Poznan University of Medical Sciences, Mickiewicza 2, Poznań, 60-834 Poland

**Keywords:** Erythrocyte indices, Diabetes mellitus, type 1, Risk assessment, Heart disease risk factors, Cardiovascular diseases

## Abstract

**Introduction:**

We evaluated the association between Red Cell Distribution Width (RDW) and predicted 10-year cardiovascular disease (CVD) risk, as estimated by the Steno Type 1 Risk Engine (ST1RE), in individuals with type 1 diabetes (T1D).

**Methods:**

We conducted a retrospective analysis of 342 adults with T1D duration > 5 years, (163 women, 179 men) from a tertiary Diabetes Center electronic database. Participants were stratified into tertiles of RDW: Group 1 (G1: < 12.6), Group 2 (G2: 12.6–13.2), and Group 3 (G3: >13.2).

**Results:**

Higher RDW was associated with older age and longer diabetes duration. The prevalence of microvascular complications did not differ across RDW tertiles. Predicted 10-year CVD risk (ST1RE 10Y) increased with higher RDW: median (IQR) 4.5 (3.2–6.1) in G1, 4.5 (2.9–7.2) in G2, and 6.2 (3.5–12.0) in G3 (*p* < 0.01). In multiple linear regression, RDW was positively associated with ST1RE 10Y, (β = 1.13;95% CI, 0.57–1.70; *p* < 0.01; R^2^ = 0.36). In multivariable logistic regression, RDW was independently associated with moderate/high versus low ST1RE 10Y risk (OR = 1.87;95%CI, 1.28–2.75; *p* = 0.001). Models were adjusted for presence of hypertension, dyslipidemia, diabetic kidney disease, BMI value and hsCRP concentration.

**Conclusion:**

Our results suggest that RDW is independently associated with predicted 10-year CVD risk in individuals with T1D. These findings support RDW as a potential marker for cardiovascular risk stratification. However, external validation is required before clinical application.

**Supplementary Information:**

The online version contains supplementary material available at 10.1007/s00592-025-02615-y.

## Introduction

Despite advances in the management of cardiovascular disease (CVD) risk factors and improved glycemic control, CVD remains the leading cause of mortality among individuals with type 1 diabetes (T1D) [1–3]. Accurate identification of CVD risk is therefore critical but remains insufficiently addressed in this population.

The Steno T1 Risk Engine (ST1RE) is a predictive tool developed to estimate the 5- and 10-year risk of non-fatal and fatal CVD- including ischemic heart disease, stroke, and peripheral vascular disease- in individuals with T1D. This risk calculator is based on comprehensive clinical data from approximately 5.000 T1D patients treated at the Steno Diabetes Center, Copenhagen, linked to national registry data, and validated using the Funen Diabetes Database [4].

Researchers using the European Society of Cardiology (ESC) risk stratification and the 10-year cardiovascular risk predicted by the ST1RE tool found that nearly half of individuals with type 1 diabetes (T1D) without prior cardiovascular disease (CVD) fall into the *very high-risk* category. However, when they applied the ST1RE algorithm, none of the patients younger than 35 years and only about 12% of those aged 35 years or older met the criteria for this highest risk group [5]. Researchers have also used ST1RE to examine how interventions such as sodium–glucose cotransporter-2 (SGLT2) inhibitors reduce cardiovascular risk in people with T1D [6].

The ESC approach identifies individuals with the greatest CVD risk effectively but loses predictive accuracy in lower-risk categories. In contrast, the ST1RE model provides stronger discrimination across all levels of risk [7].

Multiple factors contribute to the development of coronary artery disease (CAD) in T1D, including traditional cardiovascular risk factors, insulin resistance, genetic susceptibility, and chronic inflammation. Clinicians routinely measure red cell distribution width (RDW) — a hematologic marker that reflects variation in red blood cell size (anisocytosis)—as part of a complete blood count. Physicians initially used RDW to evaluate anemia [8, 9], but recent studies show that higher RDW levels predict worse outcomes in CAD, heart failure, and metabolic syndrome [9, 10]. Elevated RDW reflects chronic low-grade inflammation, which drives cardiovascular disease in diabetes [8, 9, 11]. Its role in T1D, however, has not been fully evaluated. We therefore aimed to investigate the association between RDW and 10-year predicted CVD risk as estimated by the Steno Type 1 Risk Engine (ST1RE 10Y) in individuals with type 1 diabetes.

## Materials and methods

We conducted a retrospective study using clinical records from individuals with T1D from the electronic database of the Department of Internal Medicine and Diabetology, Poznan University of Medical Sciences (The Bioethical approval no 560/25). Inclusion criteria were: confirmed diagnosis of T1D based on WHO criteria and autoantibody positivity [against glutamic acid decarboxylase (anty-GAD), or islet antigen-2 (anty-IA-2), or pancreatic islet cells antibodies (ICA), or anti-ZnT8 antibodies (ZnT8A)], diabetes duration longer than 5 years, age 18–50 years. We excluded participants with diagnosed hematologic disorders, defined according to local laboratory reference ranges as hemoglobin < 11.1 g/dL (< 6.9 mmol/L) in women or < 13.7 g/dL (< 8.5 mmol/L) in men, or treatment of anemia during the past 3 months, because anemia significantly affects RDW values and could therefore confound its association with cardiovascular risk [12].

We also excluded people with chronic kidney disease with an estimated glomerular filtration rate (eGFR) below < 60 ml/min/1.73 m², macrovascular complications of diabetes (peripheral artery disease, ischemic heart disease, or stroke), acute inflammation [defined as serum high-sensitivity C-reactive protein (hsCRP) > 10 mg/L, white blood cell count (WBC) > 15 × 10⁹/l, or erythrocyte sedimentation rate (ESR) > 30 mm/h], and hepatic dysfunction (alanine or aspartate aminotransferase > 2x upper limit). RDW was expressed as the coefficient of variation (RDW-CV), calculated as:

$${\mathrm{RDW}} - {\text{CV }}={\text{ }}[({\mathrm{L2}}\, - \,{\mathrm{L1}})/({\mathrm{L2}}\,+\,{\mathrm{L1}})] \times {\mathrm{1}}00$$

where L1 = MCV − 1 SD and L2 = MCV + 1 SD.

RDW-CV values were obtained using the SYSMEX XN 550 analyzer (Sysmex Corporation, Japan) [13]. Glycated hemoglobin (HbA_1_c) levels were assessed using high-performance liquid chromatography. Serum lipid profile, including total cholesterol, LDL-cholesterol, HDL-cholesterol, and triglycerides, was measured after an overnight fast using enzymatic methods (BioMerieux, Lyon, France). High-sensitivity C-reactive protein (hsCRP) was quantified by turbidimetric immunoassay (Roche/Hitachi, Cobas). Chronic diabetes complications were diagnosed according to established guidelines. Peripheral neuropathy was tested using sensory assessments and reflex testing, diagnosing neuropathy if two or more specific criteria were met. Diabetic retinopathy was diagnosed and classified using fundus examinations with detailed photographic documentation [14]. Diabetic kidney disease was diagnosed based on serum creatinine, eGFR, and albuminuria, with progression classified according to the National Kidney Foundation Disease Outcomes Quality Initiative criteria [15, 16]. More detailed descriptions of the evaluation of diabetic complications can be found in our previous report [17].

Predicted 10-year CVD risk was calculated using the ST1RE, accessed via the official online platform (https://steno.shinyapps.io/T1RiskEngine/). Required input variables included age, sex, diabetes duration, systolic blood pressure, glycated haemoglobin (HbA₁c), smoking status, LDL cholesterol, regular exercise (3.5 h/week), albuminuria status, and eGFR. All values were obtained from clinical and laboratory records at the time of assessment.

Participants were classified into risk categories according to thresholds used in the original Steno Type 1 Risk Engine (ST1RE) derivation study and in line with current European Society of Cardiology (ESC) guidelines for cardiovascular prevention. Individuals with a predicted 10-year cardiovascular risk of < 10% were classified as having low risk, those with risk between 10 and 20% as moderate risk, and those with risk >20% as high risk. These categories were applied consistently throughout the analysis [18, 19].

Participants were stratified into three groups based on RDW tertiles: Group 1 (G1: RDW < 12.6%), Group 2 (G2: RDW 12.6–13.2%), and Group 3 (G3: RDW > 13.2%).

### Statistical analysis

For statistical analysis, we used the statistical package Dell Statistica, version 13.1 (Dell Inc. 2016, www.software.dell.com) and MedCalc Statistical Software version 23.0.9 (MedCalc Software Ltd, Ostend, Belgium; https://www.medcalc.org; 2024). Normality was tested using the Kolmogorov-Smirnov test with Lilliefors correction. Since the data were not normally distributed, we used nonparametric tests for further analysis. Data were presented as counts and percentages or as medians and interquartile ranges (IQR). To compare numerical variables across subgroups, the Kruskal-Wallis test was used, followed for statistically significant results by post-hoc Dunn’s test. Chi-squared test was used to evaluate differences in categorical data, and, for statistically significant results, with subsequent pairwise comparison with Bonferroni correction. Multiple linear regression was applied to assess the association between RDW and ST1RE 10Y, while multivariable logistic regression was used to evaluate this association when ST1RE 10Y was analyzed as a dichotomous variable (moderate/high vs. low risk). Both regression models included the following covariates: presence of hypertension, dyslipidemia, diabetic kidney disease, BMI value and hsCRP concentration. The analyses were conducted on a complete case dataset. A p-value of < 0.05 was considered statistically significant.

## Results

A total of 342 participants were included (163 women and 179 men). The median age was 34 years (IQR 28–39), with a median diabetes duration of 14 years (IQR 10–20). Diabetic retinopathy was diagnosed in 122 participants (35.6%), kidney disease in 45 (13.1%), and neuropathy in 56 (16.3%) (Table 1).

**Table 1:** Characteristic of study group

Variable	Study group (n = 342)
Sex (female/male) (n) (%)	163 (48)/179 (52)
Age (years)	34 (28–39)
Duration of diabetes (years)	14 (10–20)
BMI (kg/m^2^)	25.1 (22.6–28.0)
SBP (mmHg)	127 (120–134)
DBP (mmHg)	80 (70–85)
Daily insulin dose (U/kg body weight)	0.5 (0.4–0.6)
Hb_A1c_ (mmol/mol)	61 (52–73)
TCH (mmol/L)	4.9 (4.4–5.5)
TG (mmol/L)	1.0 (0.8–1.3)
LDL cholesterol (mmol/L)	2.8 (2.3–3.4)
HDL cholesterol (mmol/L)	1.7 (1.4–2.0)
hsCRP (mg/L)	1.13 (0.61–2.49)
eGFR (MDRD) (ml/min/1.73m^2^)	97.8 (88.2–110.0)
ST1RE 10Y (%)	4.7 (3.1–7.8)
WBC (× 10^9^/L)	6.4 (5.6–7.5)
Hemoglobin (mmol/L)	8.9 (8.3–9.6)
Platelet count (× 10^9^/l)	248 (211–294)
RDW	12.9 (12.4–13.4)
Retinopathy (n) (%)	122 (35.6)
Diabetic Kidney Disease (n) (%)	45 (13.1)
Neuropathy (n) (%)	56 (16.3)
Smoking (past and current) (n) (%)	84 (24.6)
Hypertension (n) (%)	106 (31.0)
Statin use (n) (%)	43 (12.6)

*BMI*, body mass index; *HbA1c*, glycated haemoglobin; *TCH*, total cholesterol; *TG*, triglycerides; *LDL*, low density lipoproteins; *HDL*, high density lipoproteins; *eGFR*, glomerular filtration rate estimated using Modification of Diet in Renal Disease (MDRD) study equation; ST1RE 10Y, 10-year Steno Type 1 Risk; *SBP*, systolic blood pressure; *DBP*, diastolic blood pressure; *WBC*, white blood cell count; *hsCRP*, high-sensitivity C-reactive protein; *RDW-CV*, red blood cell distribution width. Values are presented as median (IQR, 25th-75th percentile) if no otherwise specified

Comparison across RDW tertiles showed that participants in the highest RDW group (G3) were older and had longer diabetes duration. HbA1c was lowest in G2 and highest in G3 [7.4 (6.7–8.6) vs. 7.9 (7.1–8.9), *p* = 0.040]. The lowest eGFR values were observed in G3. Prevalence of microvascular complications and statin use did not differ between tertiles. ST1RE 10Y increased with higher RDW levels, from 4.5 (3.2–6.1) in G1 to 6.2 (3.5–12.0) in G3 (*p* < 0.01) (Table 2).

**Table 2:** Comparison of groups according to RDW tertiles

Variable	Group1 (G1) (RDW < 12.6)	Group2 (G2) (12.6 ≤ RDW ≤ 13.2)	Group3 (G3) (RDW > 13.2)	p
N	107	127	108	–
Sex (female/male) (n) (%)	48(45)/59(55)	55 (43)/72 (57)	60(56)/48(44)	0.13
Age (years)	32 (27–37)	34 (28–39)	36 (31–41)	< 0.001*
Duration of diabetes (years)	14 (8–18)	14 (9–19)	15 (11–21)	**0.046**
BMI (kg/m^2^)	24.9 (22.8–27.4)	24.8 (23.0–28.0)	25.6 (22.5–28.4)	0.55
SBP (mmHg)	130 (120–133)	124 (120–130)	130 (120–140)	0.06
DBP (mmHg)	80 (70–80)	80 (70–85)	80 (70–85)	0.05
Daily insulin dose (U/kg body weight)	0.5 (0.4–0.6)	0.5 (0.4–0.6)	0.4 (0.4–0.6)	0.37
Hb_A1c_ (mmol/mol)	61 (51–73)	57 (50–70)	63 (54–74)	**0.040** ^**#**^
TCH (mmol/L)	4.9 (4.4–5.5)	4.9 (4.5–5.4)	5,1 (4.5–5.7)	0.36
TG (mmol/L)	1.0 (0.7–1.2)	1.1 (0.8–1.4)	1.0 (0.7–1.3)	0.24
LDL cholesterol (mmol/L)	2.7 (2.1–3.5)	2.8 (2.4–3.3)	2.9 (2.5–3.6)	0.23
HDL cholesterol (mmol/L)	1.7 (1.4–1.9)	1.7 (1.4–2.1)	1.7 (1.4–2.0)	0.91
WBC (× 10⁹/l)	6.04 (5.30–7.15)	6.62 (5.58–7.77)	6.59 (5.69–7.63)	0.033
hsCRP (mg/L)	1.01 (0.39–2.37)	1.13 (0.64–2.57)	1.38 (0.62–2.93)	0.20
eGFR (MDRD) (ml/min/1.73m^2^)	102 (91–114)	99 (86–109)	93 (86–106)	0.006*
ST1RE 10Y (%)	4.5 (3.2–6.1)	4.5 (2.9–7.2)	6.2 (3.5–12.0)	0.007* ^**#**^
Retinopathy (n) (%)	31 (29.0)	45 (35.0)	46 (42.6)	0.11
Diabetic Kidney Disease (n) (%)	9 (8.4)	15 (11.8)	21 (19.4)	0.06
Neuropathy (n) (%)	14 (13.0)	19 (15.0)	23 (21.3)	0.22
Smoking past and current (n) (%)	25 (23.0)	30 (23.6)	29 (26.8)	0.80
Hypertension (n) (%)	30 (28.0)	35 (27.6)	41 (38.0)	0.17
Statin use (n) (%)	15 (14.0)	9 (7.1)	19 (17.6)	0.046

Bold value indicate statistically significant p < 0.05

*BMI*, body mass index; *HbA1c*, glycated haemoglobin; *TCH*, total cholesterol; *TG*, triglycerides; *LDL*, low density lipoproteins; *HDL*, high density lipoproteins; *eGFR*, glomerular filtration rate estimated using Modification of Diet in Renal Disease (MDRD) study equation; ST1RE 10Y, 10-year Steno Type 1 Risk; SBP, systolic blood pressure; DBP, diastolic blood pressure; WBC, white blood cell count; , high-sensitivity C-reactive protein; RDW, red blood cell distribution width. Values are presented as median (IQR, 25th-75th percentile) if no otherwise specified

*Significant difference between groups 1 and 3

^#^Significant difference between groups 2 and 3

In multiple linear regression, RDW was positively associated with ST1RE 10Y, (β = 1.13;95% CI, 0.57–1.70; *p* < 0.01; R^2^ = 0.36), independent from presence of hypertension, dyslipidemia, diabetic kidney disease, BMI value and hsCRP concentration.

Multivariable logistic regression identified RDW as factor significantly associated with elevated predicted ST1RE 10Y (moderate or high versus low 10-year risk): (OR = 1.87;95%CI, 1.28–2.75; *p* = 0.001), after adjustment for all covariates (Full regression results are provided in electronic supplementary material – Supplementary Tables 1 and 2).

## Discussion

We found that RDW was positively associated with the ST1RE 10Y, independent of presence of hypertension, dyslipidemia, diabetic kidney disease, BMI value and hsCRP concentration. These findings support the potential role of RDW as a biomarker of cardiovascular risk in individuals with T1D. Elevated RDW has previously been linked to a range of acute and chronic cardiovascular conditions, including acute coronary syndrome, ischemic cerebrovascular disease, peripheral artery disease, heart failure, atrial fibrillation, and hypertension [20]. In a real-world cohort of patients, Talarico et al. found that higher RDW values were independently associated with increased all-cause mortality and adverse cardiovascular events, proposing RDW as a prognostic marker in patients with cardiovascular disease [21]. Similarly, Wen et al. reported a significant association between elevated RDW and advanced subclinical atherosclerosis, reflected by increased intima-media thickness and carotid plaques [22]. Data from the CHARM trial further identified RDW as a strong and independent predictor of all-cause mortality and adverse cardiovascular outcomes in patients with heart failure [23]. The biological mechanisms underlying these associations remain incompletely understood. Conditions such as chronic inflammation and hemolytic anemia, which affect bone marrow function, can lead to the release of immature and morphologically abnormal erythrocytes, thereby increasing RDW [24]. In the NHANES study, individuals with elevated RDW had a higher 10-year risk of coronary heart disease, even after adjusting for cardiovascular risk factors and conditions related to anemia and vitamin B deficiency [12]. While most prior studies have focused on RDW in the general population or in individuals with type 2 diabetes, our findings provide evidence that anisocytosis may also capture unrecognized cardiovascular risk in type 1 diabetes, a population in which traditional risk calculators frequently underestimate true cardiovascular burden.

Further evidence from large-scale prospective studies underscores the prognostic potential of RDW. Both the Malmö Diet and Cancer Study and the Tromsø Study, which together included more than 25,000 initially healthy individuals aged 40–70 years, reported that elevated RDW was independently associated with risk of acute myocardial infarction, even after adjusting for several cardiovascular risk factors [25]. In the Malmö cohort, participants in the highest RDW quartile at baseline had a 1.8-fold increased risk of fatal acute coronary events over 14 years of follow-up [26]. Prospective evaluation of RDW in T1D populations is therefore warranted to determine its predictive utility.

Our findings align with previous research demonstrating a consistent association between elevated RDW and diabetes-related microvascular complications, particularly nephropathy. In a cohort of individuals with type 2 diabetes and proliferative retinopathy, Magri et al. observed an independent association between RDW and nephropathy after adjustment for multiple confounders, whereas no significant associations were found with neuropathy or peripheral arterial disease [27]. In T1D, Zhang et al. reported significantly higher RDW values in children with microalbuminuria compared to those without, and multivariable analyses confirmed RDW as an independent predictor of early renal impairment [28]. Likewise, Gu and Xue demonstrated in 396 patients with diabetic chronic kidney disease that RDW correlated positively with disease severity across CKD stages and remained a significant predictor after adjustment for demographic and clinical variables. Notably, the strongest association was observed in patients with intermediate diabetes duration and suboptimal HbA_1c_ control [29].

Mechanistically, elevated RDW has been associated with chronic low-grade inflammation, oxidative stress, impaired iron metabolism, and bone marrow responsiveness to erythropoietin. These processes can disrupt erythrocyte maturation, shorten red blood cell lifespan, and increase anisocytosis [30]. These alterations not only mirror the inflammatory and oxidative milieu underlying diabetic nephropathy but also overlap with the pathophysiological mechanisms driving atherosclerosis and cardiovascular disease. Indeed, RDW has emerged as a prognostic marker for a wide range of cardiovascular outcomes, including myocardial infarction, heart failure, stroke, and peripheral artery disease [31]. The independent association of RDW with ST1RE risk, after adjustment for HbA_1c_, hypertension, diabetic kidney disease, and LDL cholesterol, suggests that RDW may reflect additional pathophysiological processes—such as low-grade inflammation or altered erythropoiesis—not fully accounted for by conventional risk factors.

Beyond cardiovascular disease, RDW may also hold prognostic value for the development of diabetes. Interestingly, lower RDW has been linked to a higher incidence of diabetes mellitus, potentially reflecting reduced red blood cell survival and lower HbA1c levels due to shorter glucose exposure [32]. RDW could thus enhance risk stratification for diabetes and its complications. For instance, higher RDW levels have been associated with diabetic retinopathy [33], while Magri et al. reported associations between RDW and both neuropathy and diabetic kidney disease [27]. Similarly, Zhang et al. demonstrated that children with albuminuria exhibit higher RDW [28]. Although the role of anisocytosis in the pathogenesis of cardiovascular diseases remains uncertain, accumulating evidence suggests that the clinical application of RDW may extend beyond its traditional role in hematological disorders [34]. Our study demonstrates that a connection between RDW and cardiovascular risk is present also in individuals with type 1 diabetes. While prior evidence in this population is limited, we extend current knowledge by evaluating this relationship using the ST1RE risk model in a well-characterized T1D cohort. Multiple logistic regression analysis further revealed that longer diabetes duration, presence of hypertension, diabetic kidney disease, and higher RDW were all significantly associated with an increased ST1RE 10Y. RDW may be considered a useful marker for predicting cardiovascular events and all-cause mortality in individuals with diabetes [12, 35, 36]. Given that RDW is an inexpensive and widely available hematological parameter, its association with higher ST1RE-predicted cardiovascular risk highlights a potential role in improving risk stratification in T1D.

This study has limitations. The retrospective, cross-sectional design precludes causal inference and may be subject to selection bias, as participants were drawn from a single tertiary care center and may not fully represent the broader T1D population. Although we adjusted for key clinical variables, residual confounding from unmeasured factors—such as dietary status, iron metabolism parameters, inflammatory markers beyond hsCRP, or socioeconomic variables—cannot be excluded. Furthermore, because we relied on calculated ST1RE risk scores, confirmation of a direct association between RDW and actual cardiovascular events or all-cause mortality requires prospective studies with adjudicated outcomes. The restriction of our study population to adults aged 18–50 years and the exclusion of individuals with eGFR < 60 mL/min/1.73 m² may limit the generalizability of our findings. Finally, the retrospective design does not rule out the possibility that abnormal RDW values may reflect unrecognized comorbidities (e.g., incipient anemia), that are unrelated to the outcomes assessed in this analysis.

## Conclusions

Our findings suggest that RDW is associated with ST1RE-predicted 10-year cardiovascular risk in individuals with type 1 diabetes. Since ST1RE reflects predicted rather than observed risk, further external validation in prospective cohorts is needed before this can be translated into clinical practice.

## Supplementary Information

Below is the link to the electronic supplementary material.

Supplementary Material 1

Supplementary Material 2

## Data Availability

The data that support the findings of this study are available from the corresponding authors upon reasonable request.

## Abbreviations

RDW: Red Cell Distribution Width
RDW-CV: Red Blood Cell Distribution Width – Coefficient of Variation
ST1RE: Steno Type 1 Risk Engine
T1D: Type 1 diabetes
CVD: Cardiovascular disease
CAD: Coronary artery disease
eGFR: Estimated glomerular filtration rate
HbA_1_c: Glycated hemoglobin
hsCRP: High-sensitivity C-reactive protein
