# Supplementary material for: Red cell distribution width positively correlates with 10-year risk of cardiovascular disease among people with type 1 diabetes as assessed by the Steno Type 1 Risk Engine
Source: Acta Diabetol. 2025 Nov 24;63(2):277–83. doi: 10.1007/s00592-025-02615-y (PMC12956950; doi:10.1007/s00592-025-02615-y)
Supplement: Supplementary file 1 — Supplementary Material 1 [file 592_2025_2615_MOESM1_ESM.docx]

Supplementary material

Supplementary table 1. Association between RDW and ST1RE 10Y. Multiple linear regression model (R^2^=0.36).

| Variable | Coefficient (β) | Std. Error | 95% CI | P |
| --- | --- | --- | --- | --- |
| RDW | 1,1347 | 0,2894 | 0,5654 – 1,7039 | 0,0001 |
| BMI | 0,003326 | 0,06827 | -0,1310 – 0,1376 | 0,9612 |
| Dyslipidemia | 2,9086 | 0,6802 | 1,5704 – 4,2467 | <0,0001 |
| Hypertension | 2,9051 | 0,6030 | 1,7188 – 4,0914 | <0,0001 |
| Diabetic Kidney Disease | 5,2604 | 0,7621 | 3,7612 – 6,7595 | <0,0001 |
| hsCRP [mg/L] | 0,0003794 | 0,1336 | -0,2623 – 0,2631 | 0,9977 |

Supplementary table 2. Association between RDW and ST1RE 10Y (low versus intermediate or high risk). Multiple logistic regression model.

| Variable | Coefficient | Std. Error | Wald χ^2^ | P | OR | 95% CI |
| --- | --- | --- | --- | --- | --- | --- |
| RDW | 0,62757 | 0,19534 | 10,3213 | 0,0013 | 1,8731 | 1,2772 – 2,7468 |
| BMI | -0,042342 | 0,049208 | 0,7404 | 0,3895 | 0,9585 | 0,8704 – 1,0556 |
| Dyslipidemia | 1,20082 | 0,39690 | 9,1537 | 0,0025 | 3,3228 | 1,5264 – 7,2336 |
| Hypertension | 1,72837 | 0,39184 | 19,4559 | <0,0001 | 5,6315 | 2,6126 – 12,1385 |
| Diabetic Kidney Disease | 1,51734 | 0,41508 | 13,3627 | 0,0003 | 4,5601 | 2,0214 – 10,2873 |
| hsCRP [mg/L] | -0,0071629 | 0,090484 | 0,006267 | 0,9369 | 0,9929 | 0,8315 – 1,1855 |
