# Supplementary material for: Red cell distribution width positively correlates with 10-year risk of cardiovascular disease among people with type 1 diabetes as assessed by the Steno Type 1 Risk Engine
Source: Acta Diabetol. 2025 Nov 24;63(2):277–83. doi: 10.1007/s00592-025-02615-y (PMC12956950; doi:10.1007/s00592-025-02615-y)
Supplement: Supplementary file 2 — Supplementary Material 2 [file 592_2025_2615_MOESM2_ESM.pdf]

POZNAN UNIVERSITY OF MEDICAL SCIENCES
DEPARTMENT OF INTERNAL MEDICINE AND DIABETOLOGY

Mickiewicza 2
60-834 Poznan
Poland

phone: + 48 612245270
fax: + 48 612245445
e-mail: kldiab@raszeja.poznan.pl

Poznan 26th July 2025

The authors:

Dariusz Naskret dnaskret@ump.edu.pl
ORCID 0000-0002-6927-7812

Pilacinski Stanisław pilacins@ump.edu.pl
ORCID 0000-0002-7271-1683

Niedzwiecki Paweł pniedzwiecki@ump.edu.pl
ORCID 0000-0002-4033-0085

Kulecki Michał 73518@student.ump.edu.pl
ORCID 0000-0002-5224-6948

Dorota Zozulinska -Ziolkiewicz dzozulinskaziolkiewicz@ump.edu.pl
ORCID 0000-0003-2995-9971

article entitled: **“Red Cell Distribution Width (RDW) Positively Correlates with 10-Year Risk of Cardiovascular Disease Among People with Type 1 Diabetes as Assessed by the Steno Type 1 Risk Engine”** declare no conflicts of interest.

Dariusz Naskret MD, PhD
